# Surround Sensing Technique for Trucks Based on Multi-Features and Improved Yolov5 Algorithm

**DOI:** 10.3390/s24072112

**Published:** 2024-03-26

**Authors:** Zixian Li, Yongtao Li, Hanyan Li, Liting Deng, Rungang Yan

**Affiliations:** 1 School of Mechanical and Automotive Engineering, Guangxi University of Science and Technology, Liuzhou 545616, China; lizx9gxust@163.com; 2 School of Automation, Guangxi University of Science and Technology, Liuzhou 545616, China; lihanyan@gxust.edu.cn; 3 Department of Electronics and Appliances, Dongfeng Liuzhou Motor Co., Ltd., Liuzhou 545616, China; denglt@dflzm.com (L.D.); yanrg@dflzm.com (R.Y.)

**Keywords:** target location, SIFT, corner feature, image mosaic, YOLOv5

## Abstract

The traditional rearview mirror method cannot fully guarantee safety when driving trucks. RGB and infrared images collected by cameras are used for registration and recognition, so as to achieve the perception of surroundings and ensure safe driving. The traditional scale-invariant feature transform (SIFT) algorithm has a mismatching rate, and the YOLO algorithm has an optimization space in feature extraction. To address these issues, this paper proposes a truck surround sensing technique based on multi-features and an improved YOLOv5 algorithm. Firstly, the edge corner points and infrared features of the preset target region are extracted, and then a feature point set containing the improved SIFT algorithm is generated for registration. Finally, the YOLOv5 algorithm is improved by fusing infrared features and introducing a composite prediction mechanism at the prediction end. The simulation results show that, on average, the image stitching accuracy is improved by 17%, the time is reduced by 89%, and the target recognition accuracy is improved by 2.86%. The experimental results show that this method can effectively perceive the surroundings of trucks, accurately identify targets, and reduce the missed alarm rate and false alarm rate.

## 1. Introduction

With the growth of the automotive industry, image processing- and recognition-related technologies have been applied to vehicles, and the experience of driving has become better. For example, 360-degree panoramic technology effectively assists drivers in perceiving the surroundings of their vehicle and reduces the possibility of accidents caused by blind spots of vision. In contrast to normal cars, trucks are larger in size, usually with external carriages, and have a large range of visual blind spots. The surroundings of trucks cannot be fully sensed only through the traditional rearview mirror method, and perception is needed to achieve safe driving. The key aspect of surround sensing technique includes image registration, matching multiple images, and target recognition of processed images, so as to ensure safe driving; the images collected and processed include RGB and infrared images.

The SIFT algorithm was proposed by Lowe in 2004 [1], and is very stable against changes in illumination, affine distortion and noise, and these advantages make the algorithm very suitable for the image registration process [2]. However, the algorithm also has certain shortcomings. Due to the need to extract 128-dimensional features and the long computing time, it does not have advantages in terms of real-time performance. In view of these shortcomings, many scholars have put forward a series of improvement measures.

For the improvement of the matching algorithm, improvement methods have been proposed for redundant features by reducing features [3]. By recombining 16 pixels at key points, the 128-dimensional vector of the SIFT descriptor is transformed into the method of a reconstruction vector [4]. Other methods include feature extraction through SIFT descriptors of color-independent components for different scene classification [5], and the method of combining with HOG features [6]. The effect of ordinary dimensionality reduction is limited. On this basis, subsequent scholars have put forward some improvement measures in other fields. For example, the key point matching of the SIFT algorithm descriptor is represented as a global optimization problem, and belief propagation is adopted to provide a sub-optimal solution, thereby improving the matching accuracy [7]. Samy et al. introduced a feature extraction framework in which operations such as denoising and binarization were used for pre-processing to improve feature quality [8]. Tang et al. increased the stability factor in the scale space construction to reduce the algorithm dimensions and amount of computation required [9]. Hu et al. used the combination of the Ikebukuro algorithm and SIFT algorithm to improve the performance [10] without losing important features [11]. In addition, there is a descriptor selection algorithm based on dictionary learning, which removes redundant features and retains a small amount of features. In order to cope with different usage scenarios, the descriptors of the improved SIFT are more targeted [12].

In the field of target recognition, Qin [13] et al. performed lightweight processing on a backbone network to improve the feature extraction speed. Cui [14] et al. proposed a new mixed-space pyramid pool module to introduce a channel attention mechanism into the framework. Cao [15] et al. improved the clustering algorithm, and additional prediction heads and new feature fusion layers were added for small targets. Methods such as introducing an attention mechanism and improving the convergence speed are relatively common [16], and have improved the computation speed and accuracy to a certain extent. However, there is still the possibility of these methods being improved in terms of feature extraction and the regional localization of objects of interest. Duan et al. proposed an automatic detection solution based on panoramic imaging and deep learning object recognition for highly dynamic, low-illumination imaging environments. A YOLOv5-CCFE target detection model based on railway equipment identification is proposed. The experimental results show that the mAP@0.5 value of the YOLOv5-CCFE model reaches 98.6%, and the mAP@0.5:0.95 value reaches 68.9%. The FPS value is 158, which contributes to recognition in different lighting [17]. Li et al. customized a model based on YOLOv5 that is more suitable for vehicle detection in aerial images. First, we added an additional prediction head to detect smaller-scale targets. In addition, in order to keep the original features involved in the model training process, we introduced a bidirectional feature pyramid network to fuse the feature information at different scales [18]. The relevant literature has made certain achievements in feature fusion. Target recognition algorithms, especially YOLOv5 algorithm, are widely used in the field of automatic driving and vehicle environment perception, so their optimization is in line with the mainstream direction of research and has good application prospects. This paper improves YOLOv5 by introducing infrared and edge features, apart from the structure of the compound prediction mechanism, the accuracy of the prediction is improved; the inspiration for the improvement of methods and ideas described has been provided by the relevant literature that mentioned above.

**Figure 1:**
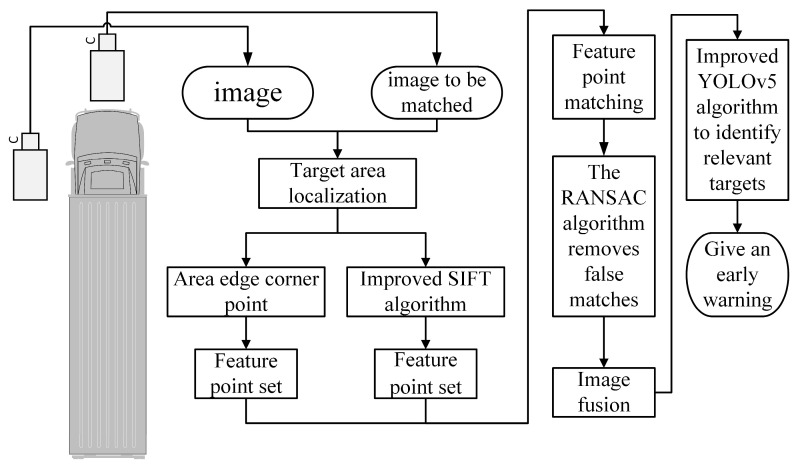
General flow chart of perceptual methods.

In this paper, for the truck environment perception system, the edge corner algorithm is adopted in an image mosaic, and a multi-feature and composite prediction mechanism is introduced into the YOLOv5 algorithm, as shown in Figure 1. Firstly, the method uses the edge corner algorithm to determine the area of the target in the image, extract the corner feature of the edge, and achieve accurate target positioning. Secondly, based on the SIFT algorithm, the feature descriptor sub-partition is optimized, and after optimization, the 128-dimensional feature descriptor is reduced to 64 dimensions, and, finally, a feature point set containing two algorithms is generated for the matching. The RANSAC algorithm is used to remove mismatching, and after the splicing image is obtained, the improved YOLOv5 algorithm is used to identify whether the image contains the expected target, so as to achieve the purpose of the surround perception of trucks. The algorithm proposed in this paper is helpful for improving the safety of truck driving. Traditional truck perception technology usually restores the surrounding environment, and there is still much room for improvement in the specific environment recognition link. The algorithm in this paper introduces the target recognition link into the perception system, and improves the image stitching part, which makes the real-time performance better and the perception ability further enhanced. It can effectively prevent the occurrence of traffic accidents.

A variety of algorithm comparison simulations and truck experiments are designed for the proposed algorithm. Compared with traditional perception methods, the proposed algorithm solves the problems of miscellaneous features, the long matching time, low feature dimension and higher matching accuracy of image stitching algorithms, and can meet the real-time requirements more easily. The YOLOv5 algorithm is improved, two indexes of image edge features and infrared features are added, and the preset data set is trained after the feature module is added to the algorithm. After training, the preset target can be identified more accurately, people or animals can be identified effectively, misidentification can be reduced, and safe driving can be ensured.

## 2. Correlation Feature Extraction

### 2.1. Target Region Feature

**Figure 2:**
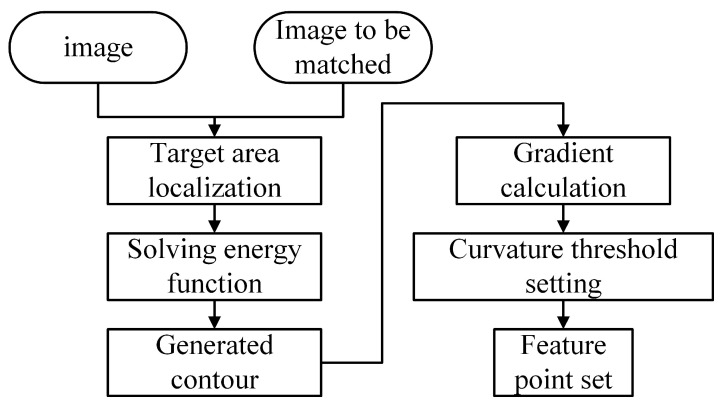
Flow chart of edge corner algorithm.

For the image collected by the body camera, the direct registration will take a lot of time to extract some feature points with low priority. The area where the vehicle and pedestrian targets are located in the image is the area of interest, and the feature extraction is more conducive to fast stitching. In this regard, this paper proposes an edge corner algorithm, as shown in Figure 2, which locates the edge contour of the truck and pedestrian targets in the image and provides a high-priority area for the subsequent extraction of feature points, so as to achieve rapid image matching and meet the requirements of time. The edge corner algorithm consists of two steps: edge contour location and corner location. The edge contour positioning is performed to first confirm the shape of the vehicles and pedestrians in the image and divide the target area, but the corner positioning is also needed to confirm the complete edge contour. The precise corner positioning adopts the LCC corner detection algorithm and determines the image shape through the image curvature.

#### 2.1.1. Edge Location

The image collected by the cameras contains a lot of information; if the relevant area is not divided, the subsequent image processing will consume a lot of time, so the division of the target area can improve the subsequent registration efficiency. For vehicles and pedestrians in the image, there is a certain color difference with the image background, and the edge contour line between the target and the background is found through the pixel difference.

Let e and f be a pair of adjacent pixels in the image, and the expression of the energy function is

(1) $$E(S)=\sum D_{e,f}(R_{e},R_{f})$$

(2) $$D_{e,f}={\left\|H_{R_{e}}(e)-H_{R_{f}}(e)\right\|}_{2}+{\left\|H_{R_{e}}(f)-H_{R_{f}}(f)\right\|}_{2}$$

where $R_{e}$ and $R_{f}$ represents the area where the pixel is located, and $H_{R_{e}}(e)$ represents the gray value of the pixel, $D_{e,f}$ describing the gray difference between two adjacent pixels. The total energy is calculated for the nodes in the image, and the difference between the vehicle and the pedestrian target and the background is large. The maximum value is found through the accumulated calculation energy, the edge contour is recorded for these nodes, and the target area is finally located.

#### 2.1.2. Corner Positioning

After the completion of the first step, more precise positioning measures are needed to make the target area to be found more accurate. The method based on image curvature is used to achieve the secondary positioning. The LCC corner detection algorithm describes the shape of related objects in the image through the curvature of the image.

Firstly, the gradient calculation of the collected images is carried out as follows:

(3) $$C=\frac{f_{xx}g_{x}^{2}-2f_{xy}g_{x}g_{y}+f_{yy}g_{x}^{2}}{g_{x}^{2}+g_{y}^{2}}$$

(4) $$\Delta C=\underset{s,t\in L(x,y)}{\max}C(s,t)-\underset{s,t\in L(x,y)}{\min}C(s,t)$$

(5) (Car,people)=∑M(ΔC≥S)

where $f_{xx}$, $f_{xy}$ and $f_{yy}$ are the second partial derivatives of the image in directions x and y, and $g_{x}$ and $g_{y}$ are the gradients in directions x and y. ΔC is the rate of change in the curvature, max is the maximum value of the field curvature, min is the minimum value of curvature, M is the corner point that meets the requirements, and S is the set threshold. The advantage of the LCC corner point algorithm is that it is fast and meets the time requirements, but there is the problem that it does not have invariance for scale change, so the SIFT feature points need to be generated to solve this problem.

### 2.2. Infrared Signature

Traditional visual perception methods have certain limitations in low-light environments. Infrared cameras are used to collect the infrared characteristics of the environment and determine whether real pedestrians and other animals appear. It can effectively protect the driver’s safety at night.

Compared with traditional visible light imaging, infrared imaging has the problems of low contrast and low resolution due to the imaging principle, so the collected infrared images are preprocessed for feature extraction. First, the infrared image is transformed linearly. Linear transformation belongs to a kind of grayscale transformation; its formula is

(6) T(x,y)=H[f(x,y)]

It can be divided into linear transformation and nonlinear transformation according to the H difference. The object, such as a pedestrian, is set as the object of interest, and linear transformation is used to highlight the object. The formula is as follows:

(7) $$L(x,y)=(\frac{m-n}{r-s}*(f(x,y)-r)+m)$$

where [m,n] is the grayscale range after transformation, and [r,s] is the grayscale range of the image before transformation.

After linear transformation, histogram equalization is used to further process the image to enhance its overall contrast [19], making the image clearer and more conducive to subsequent target recognition. The formula is as follows:

(8) $$L=T(s)=\int_{0}^{s}f_{s}(s)ds$$

(9) $$M=H(t)=\int_{0}^{t}f_{t}(t)dt$$

(10) $$t=H^{-1}(M)$$

(11) $$P_{L}(L)=P_{M}(M)$$

(12) $$t=H^{-1}(M)=H^{-1}(L)$$

where $f_{s}(s)$ and $f_{t}(t)$ are the original grayscale image and the target grayscale image, L and M are the transformation function, $P_{L}(L)$ and $P_{M}(M)$ are the probability density function, and t is the grayscale value of the target image.

## 3. Image Registration and Post-Processing

### 3.1. Improved SIFT Algorithm

The main steps of the traditional SIFT algorithm include the following: Firstly, it is necessary to establish the scale space of the image [20], and specifically construct the Gaussian function of the varying scale and the original image convolution [21]. The formulas for this are as follows:

(13) L(x,y,σ)=G(x,y,σ)∗I(x,y)

(14) $$G(x,y,\sigma )=\frac{1}{2\pi }e^{-(x^{2}+y^{2})/2\sigma^{2}}$$

where (x,y) is the pixel coordinate, σ is the scale space factor, L(x,y,σ) is the scale space of the two-dimensional image, and G(x,y,σ) is the Gaussian function of the varying scale and I(x,y) is the original image. In order to detect the key point of stability, it is also necessary to construct a Gaussian difference pyramid. The formula for this is as follows:

(15) D(x,y,σ)=L(x,y,kσ)−L(x,y,σ)

where D(x,y,σ) is the constructed Gaussian pyramid, L(x,y,kσ) and L(x,y,σ) are the image of the adjacent scale, and k is the scale factor.

In a Gaussian pyramid, the way to ensure that scale-invariant extremum points can be detected is to compare each pixel point with a total of 26 points in the same layer and in the upper and lower domains. The scale space is not continuous between each layer, and the extreme value points obtained in the first step are not necessarily the real key points. It is necessary to use the Gaussian difference pyramid function to search for interpolation in the Taylor series expansion of the scale space and remove the key points with low contrast. The formula is as follows:

(16) $$D(X)=D+\frac{\partial D^{T}}{\partial X}X+\frac{1}{2}X^{T}\frac{\partial^{2}D}{\partial X^{2}}X$$

After the key point is accurately located, the direction of the key point is determined, and the direction parameter is specified for each key point by using the gradient directionality of pixels in the neighborhood of the key point. The feature descriptor has rotation invariance. For each sampling point, L(x,y), in the window, its gradient value, m(x,y), and direction, θ(x,y), are calculated as follows:

(17) $$m(x,y)=\sqrt{{\left(L(x+1,y)-L(x-1,y)\right)}^{2}+{\left(L(x,y+1)-L(x,y-1)\right)}^{2}}$$

(18) $$\theta (x,y)=\tan^{-1}\left[\frac{L(x,y+1)-L(x,y-1)}{L(x+1,y)-L(x-1,y)}\right]$$

The histogram is used to calculate the gradient direction in a certain neighborhood of the key point, and the direction corresponding to the highest peak of the histogram is taken as the main direction of the key point.

**Figure 3:**
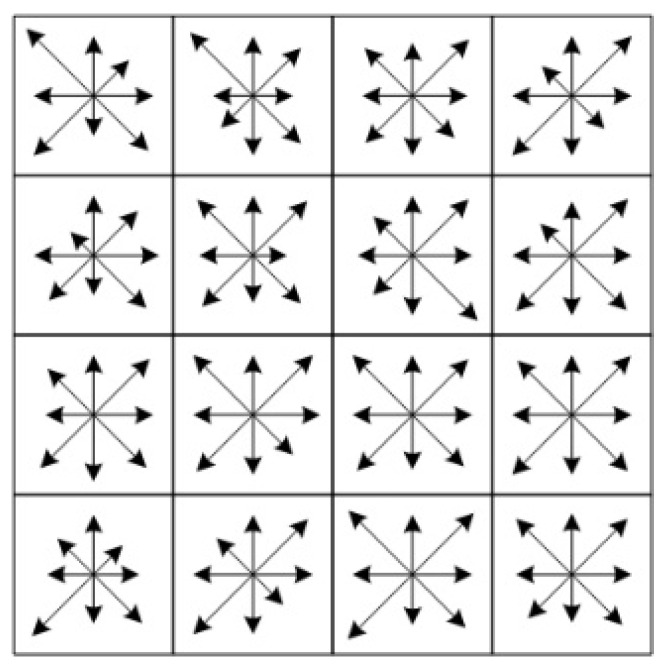
The 128-dimensional feature descriptor.

After determining the main direction, the feature descriptor can be established. The principle is to represent the detected feature points through a set of feature vectors. First, rotate the coordinate axis direction to the same direction as the feature point to ensure that the direction of the feature point remains unchanged. The region where the feature points are located is divided into 16 4 × 4 sub-regions, and each sub-region calculates the Gauss weighted cumulative values of eight gradient directions, generating 128-dimensional feature vectors, as shown in Figure 3.

**Figure 4:**
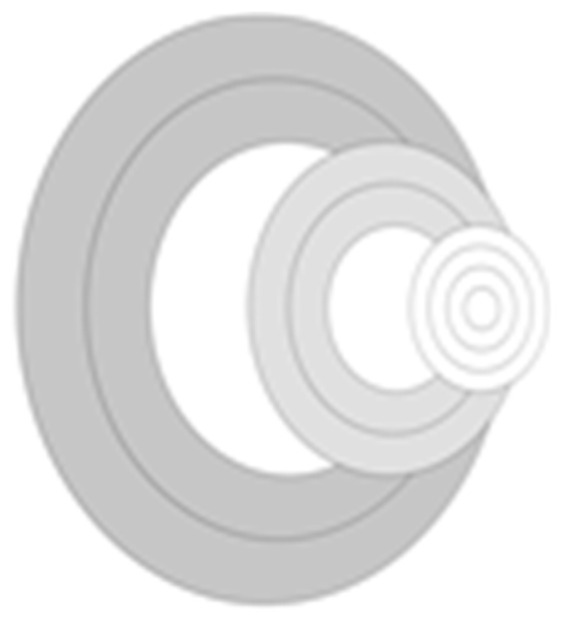
The 64-dimensional descriptor.

In this paper, the circular region is used instead of the traditional rectangular region, and the circular region has rotation invariance, which is conducive to calculating the principal direction of the feature points. For the regions around the key points, the farther the distance between them, the lower the feature correlation and the lower the influence of image registration, so different radii are assigned to the circular regions. The specific improvement is the selection of a circular area with a radius of 13 pixels, and the first area is the part near the key point, which is allocated to one circle and three concentric circle areas with smaller radii. The second region is closer to the key point and is divided into two concentric circle regions with larger radii. The third region is the region away from the key point, which is divided into two concentric circle regions with large radii. The cumulative values of the gradients in eight directions (each 45° is one direction) in each region are counted to obtain a feature descriptor of 8 × 8 = 64 dimensions, as shown in Figure 4. Finally, the effect of illumination changes on matching is reduced by normalization.

The two feature points generated in the image need to be generated into a point set for further processing. With the feature points of the SIFT algorithm as the radius, LCC corner points with pixel values within a certain range are formed into a point set as the feature points for image matching. The formula is as follows:

(19) $$D_{l}=\sqrt{{\left(x_{s}-x_{h}\right)}^{2}+{\left(y_{s}-y_{h}\right)}^{2}}$$

(20) $$T_{all}=\sum \left(s,t\right),(D_{l}\leq 2\sqrt{2})$$

where $D_{l}$ represents the distance between the feature points under the two algorithms, $\left(x_{s},y_{s}\right)$ represents the position of feature point s in the image under the SIFT algorithm, $\left(x_{h},y_{h}\right)$ represents the position of corner point h in the image obtained by the LCC corner point algorithm, and $T_{all}$ represents the formation of a new feature point set.

Compared with the traditional algorithm, the description subdimension of the algorithm is reduced by half, the time spent on the description of the feature points is reduced, and the computational complexity and amount of computation needed for the traditional algorithm are effectively reduced.

### 3.2. Image Post-Processing

In the feature point matching process, if the number of matches is large, it is necessary to use an algorithm to eliminate possible mismatching. At present, the common purification method is a random sampling consistency algorithm. This algorithm is often used in computer vision. As an iterative method, it can effectively deal with the mismatching problem of image feature points and improve the accuracy of image matching. The algorithm is divided into four steps: First, four pairs are randomly selected in the feature matching pair to form the initial point set *L*, and the single adaptation matrix is calculated, which is set as the model *M*. Secondly, the remaining matching point pairs are brought into the model *M* to calculate the error. When the error is less than the threshold, the initial point set *L* is added. The third step is to determine whether the number of points in point set *L* is set to a threshold, and when the threshold is reached, the iteration stops to obtain the optimal model *M* and point set *K*. Finally, the point set *K* is used to recalculate the single adaptation matrix.

After the image registration is completed, the image needs to be fused to obtain complete surround information, and the target recognition of the fused image is completed to achieve the goal of warning while driving. The image fusion operation includes the following steps: select one of the images as a reference, project the other images into this coordinate system, fuse the pixels with the same position in the overlapping area, retain the pixels in the non-overlapping area, fuse all the aligned images into the global plane, and adopt the weighted average method to achieve image fusion.

## 4. Improved YOLOv5 Algorithm

The feature algorithm used for the image also needs to be embedded in the target recognition algorithm. In this paper, the YOLOv5 algorithm is selected as the improvement target.

**Figure 5:**
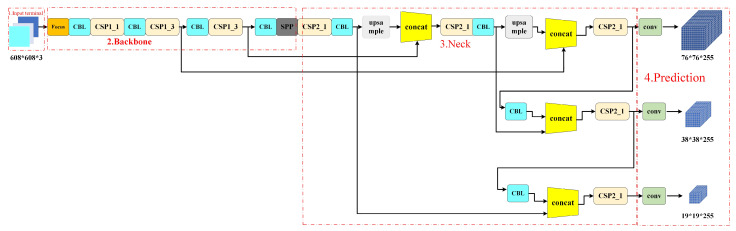
YOLOv5 algorithm structure.

The network structure of the YOLOv5 algorithm is divided into four parts: Input, Backbone, Neck and Head [22]. The input of YOLOv5 is enhanced by mosaic data, which is the same as that of YOLOv4. The main principle is as follows: select a picture and three random pictures for random cropping, and then splice them as a training set of pictures into the neural network [23]. This can not only enrich the background of the data set, improve the robustness of the system, but also reduce the loss of GPU memory and accelerate the training speed of the network. Compared with the previous generation, adaptive anchor frame calculation is added [24]. During training, the best anchor frame value of different training sets can be calculated adaptively. At the same time, an adaptive scaling strategy is adopted. The input images of different sizes are scaled into a standard size and sent into the detection network [25]. The commonly used sizes of YOLOv5 are 416 × 416 and 608 × 608. The traditional algorithm structure is shown in Figure 5.

**Figure 6:**
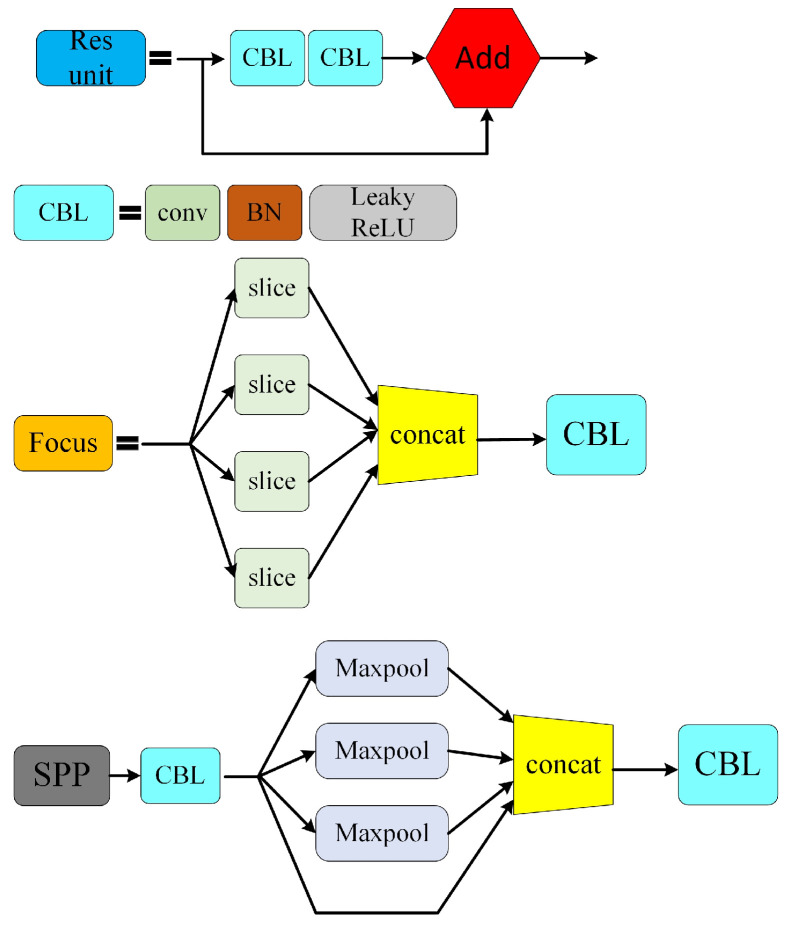
Diagram of FOCUS and other structures.

The backbone network of YOLOv5 consists of Focus, CBL, CSP, SPP and other parts [26]. The Focus structure is mainly used for slicing operations, reducing the size of the feature map by increasing the dimension of the feature map without losing any information, so that the feature number of the feature map can be increased under the premise that each piece of feature information does not change. In YOLOv5s, the 608 × 608 × 3 image is input into the Focus structure, and the feature diagram of dimensions 304 × 304 × 12 is changed by the slicing operation [27]. Then, the feature diagram of dimensions 304 × 304 × 32 is changed by a 32-kernel convolution operation. The relevant structure is shown in Figure 6. The CSP structure is already available in YOLOv4, which adopts similar design ideas as CSPNet. Compared with the previous generation of YOLO, YOLOv5 designs two CSP structures, namely, the CSP_1X and CSP_2X structures [28].

The Neck network adopts the structure of the FPN combined with PAN to strengthen the feature fusion ability of the network [29]. The FPN structure uses upsampling to improve the learning ability of the features, and PAN can better transmit strong positioning features upward.

At the output end, three detection heads are used to sample the image and generate three feature vectors of different sizes, which are used to predict the image features and generate boundary boxes and confidence [30].

There are four versions of the YOLOv5 algorithm [31]: YOLOv5s, YOLOv5m, YOLOv5l and YOLOv5x. Compared with the YOLOv5s model, the other three models all increase the depth and width of the feature map. In order to meet the requirements of the vehicle for real-time performance and speed, this paper adopts the YOLOv5s model as the target detection network.

**Figure 7:**
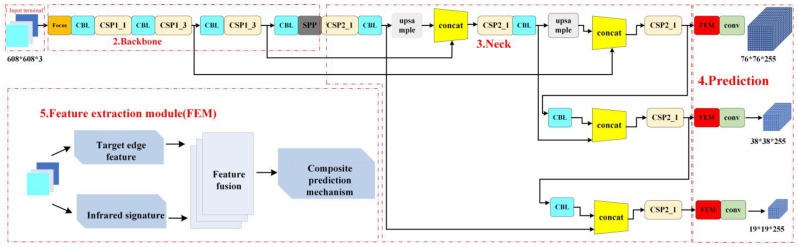
Structure diagram of YOLOv5 algorithm based on infrared edge feature.

In this paper, by adding the feature detection module, the feature algorithm is fused into the YOLOv5s algorithm, and the improved algorithm structure is shown as follows. The feature module helps in achieving the high-precision identification of objects in the region of interest. The input image is divided into two parts: traditional processing and feature module processing. The traditional processing functions by entering the input end for follow-up processing, while the improved feature module carries out feature extraction operations on the image, and the parallel processing of edge features and infrared features. The relevant formulas are described in detail in the chapter on the feature algorithm. After that, it is sent into the Conv module for late prediction processing. The improved algorithm structure is shown in Figure 7.

The loss function is used to evaluate the difference between the predicted value and the real value, and to measure whether the performance of the model meets the expected requirements. In the YOLOv5 algorithm, the loss function includes border regression loss, classification probability loss and confidence loss, and the relevant formula is as follows:

(21) $$L_{CIOU}=1-IOU+\frac{\rho^{2}(b,b^{gt})}{c^{2}}+\alpha v$$

(22) $$\alpha =\frac{v}{(1-IOU)+v}$$

(23) $$v=\frac{4}{\pi^{2}}{\left(\arctan\frac{w^{gt}}{h^{gt}}-\arctan\frac{w}{h}\right)}^{2}$$

(24) $$IOU=\frac{A\cap B}{A\cup B}$$

(25) $$G=\frac{S_{q}}{S_{k}}$$

where $\rho^{2}(b,b^{gt})$ represents the Euclidean distance between the prediction box and the actual box, w and h represent the width and height of the prediction box, $w^{gt}$ and $h^{gt}$ represent the width and height of the actual box and IOU represents the intersection ratio between the prediction box and the real box.

G is the introduced composite prediction evaluation index, $S_{q}$ is the area of the target region determined by the edge features and $S_{k}$ represents the area of the prediction box. For a prediction with a higher accuracy, the value of G should be less than 1. Through the composite prediction mechanism, the preset target can be better locked and the recognition accuracy and recognition speed can be improved.

## 5. Experiment and Result Analysis

The experimental hardware platform used in this study is the Intel(R) Core(TM) i5-12450H CPU, the frequency is 2.00 GHz, and the programming platform is Pycharm2022, which is implemented under the 64-bit Windows11 operating system using Python code. The data set used in this paper is the data set BDD100K published by the University of California, Berkeley. The data set images include the target types of cars and pedestrians. In this experiment, the data set is divided into a training set and test set according to the ratio of 8:2. In the following, we will verify whether the performance of the algorithm in this paper meets our expectations through the concatenation of multiple groups of images.

**Figure 8:**
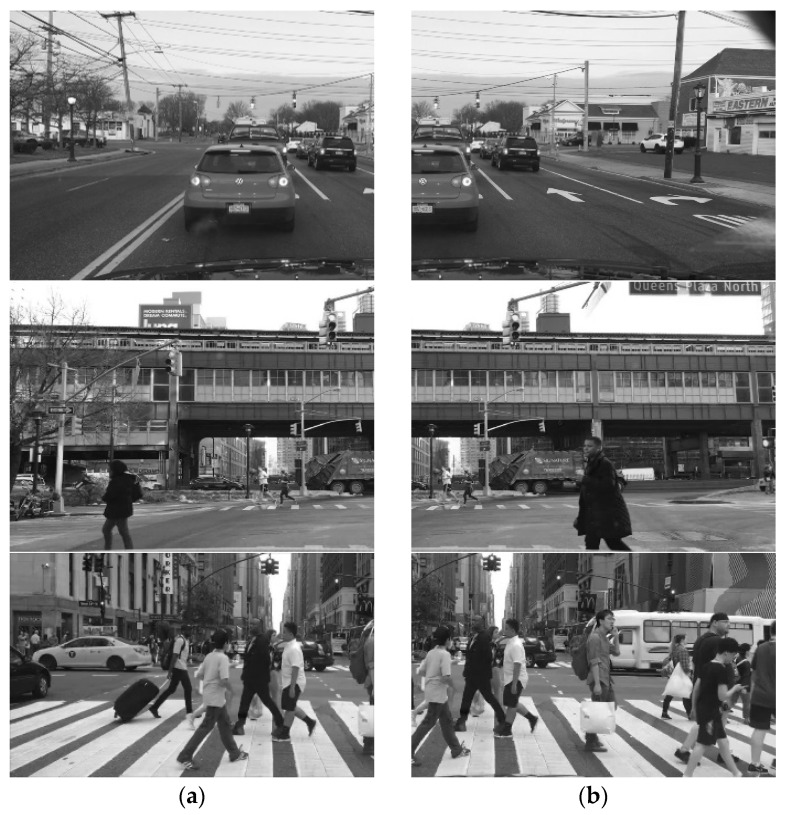
Images to be matched. (**a**) Original image; (**b**) waiting for image mosaic.

In this paper, the images in the data set are divided into three categories, namely, the pictures of road vehicles as the main body, the mixed pictures of road vehicles and pedestrians, and the original pictures of pedestrians as the main body and their images to be matched. The three groups of road condition pictures to be matched are shown below, as shown in Figure 8. The difference between the three sets of images lies in the difference in the target subjects in the images. The first set of images was taken on the road, showing the scenario of driving with other vehicles, with the vehicles as the main target; the second set of images was taken at an intersection, where both vehicles and pedestrians were present. Here, the situation was more complicated, and both were the targets; the third set of images had many rows, with pedestrians as the main target. All three sets of images are from the BDD100K data set.

### 5.1. Image Registration Experiment

**Figure 9:**
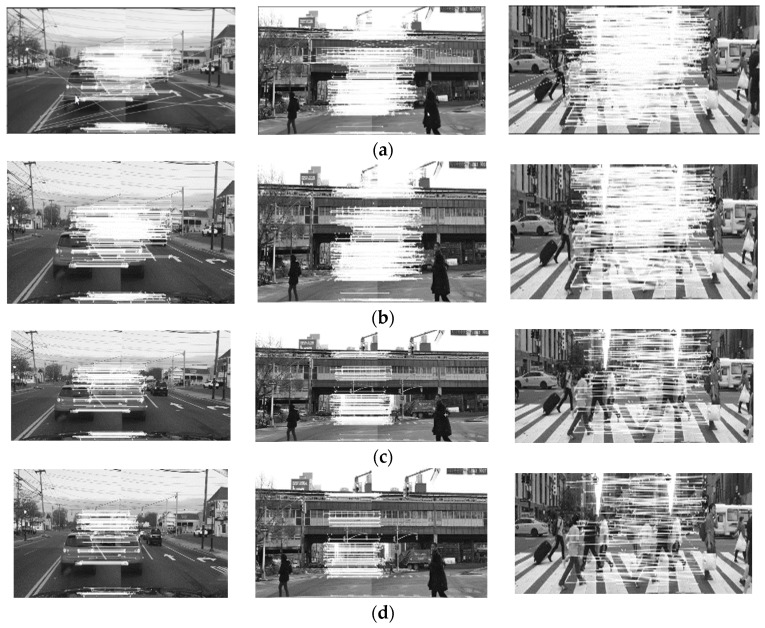
Image registration diagram: (**a**) using the traditional SIFT algorithm results in the graph; (**b**) using the improved SIFT algorithm results in the graph; (**c**) the resulting graph was obtained using the SIFT–Harris algorithm; (**d**) using this algorithm’s results in a graph.

In the first part, the improved algorithm in this paper is matched with the proposed algorithm in three road conditions. The comparison effect of the image registration of the four algorithms is shown in Figure 9. Then, the object recognition algorithm is used to simulate the spliced image obtained by the improved algorithm in this paper.

When comparing the registration effects of the four algorithms, it can be seen that the traditional SIFT algorithm has a large amount of feature matching, but there are some wrong matches. Compared with the traditional SIFT algorithm, the improved SIFT algorithm has fewer mismatches, but the overall number of matches is still at a high level, and the problem of the long matching time has not been solved. Compared with the previous two algorithms, the matching number of the SIFT–Harris algorithm is reduced, but there is still a certain amount of improvement needed before the goal of strong timeliness performance is reached. Compared with the other algorithms, the proposed algorithm reduces the dimensions of feature description, reduces the number of matches, consumes less time for feature matching, and has a certain guarantee in matching accuracy.

Table 1 shows the specific data after the simulation of the four algorithms, where NMP represents the logarithm of matching, MA represents the correct matching rate, and T represents the time taken for image matching, in seconds.

The experimental data of the algorithm in this paper under the three groups of pictures are as follows: The logarithm of the matching pairs of the first group of pictures is 164, the matching accuracy is 96.8%, and the matching time is 0.9 s. The matching logarithm of the second group of pictures is 243 pairs, the matching accuracy is 95.1%, and the matching time is 1.55 s. The third group of images was paired 179 times, the matching rate was 97.6%, and the matching time was 1.3 s.

According to the above data, compared with the traditional SIFT algorithm, on average, the matching logarithm of the proposed algorithm is reduced by 80.8%, the matching accuracy is increased by 26.4%, and the matching time is reduced by 92.4%. Compared with the improved SIFT algorithm, on average, the matching logarithm of the proposed algorithm decreases by 79.9%, the matching accuracy increases by 15.1%, and the matching time decreases by 90.1%. Compared with the SIFT–Harris algorithm, on average, the matching logarithm of the proposed algorithm is reduced by 60.4%, the matching accuracy is increased by 10.2%, and the matching time is reduced by 80.2%.

**Figure 10:**
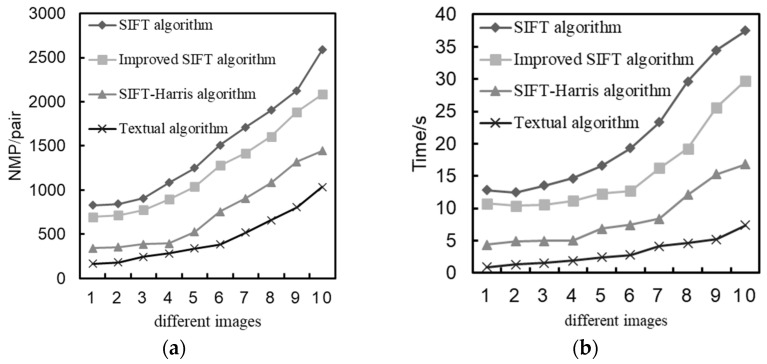
Performance comparison of four algorithms: (**a**) Comparison of the number of matching pairs; (**b**) comparison of algorithm matching time.

The algorithm in this paper reduces the total number of feature matches, improves the matching success rate, and has a reliable stitching accuracy in the three scenarios, which can ensure matching and meet the real-time requirements of automobiles. Figure 10 shows the simulation results of the four algorithms for multiple images. An abscissa from 0 to 10 represents the complexity of the scene and the number of feature points in turn, where an abscissa of 10 represents the most complex scene and the largest number of feature points. As shown in Figure 10, the different images in the horizontal coordinate refer to ten images randomly selected from the data set, which are compared by the algorithm performance and finally displayed. Compared with other comparison algorithms, the improved algorithm exhibits a significantly improved performance in terms of the matching time and the number of matching points, effectively reducing the matching logarithm and the matching time of the complex scenes.

### 5.2. Object Recognition Experiment

The data set used in this paper is a published Berkeley University data set. The data set is divided into a training set, test set and verification set according to the ratio of 8:1:1, and the data set is labeled in the YOLO format. For the experimental performance of the model, evaluation indicators are used to measure the performance, and the average precision mean (mAP), accuracy rate and recall rate are used as evaluation indicators. The relevant formulas are as follows:

(26) $$Precision=\frac{TP}{TP+FP}$$

(27) $$Recall=\frac{TP}{TP+FN}$$

where Precision is the accuracy rate, Recall is the recall rate, TP is the number of positive samples correctly identified by the model, FP is the number of false samples incorrectly identified by the model and FN is the number of samples omitted by the model.

**Figure 11:**
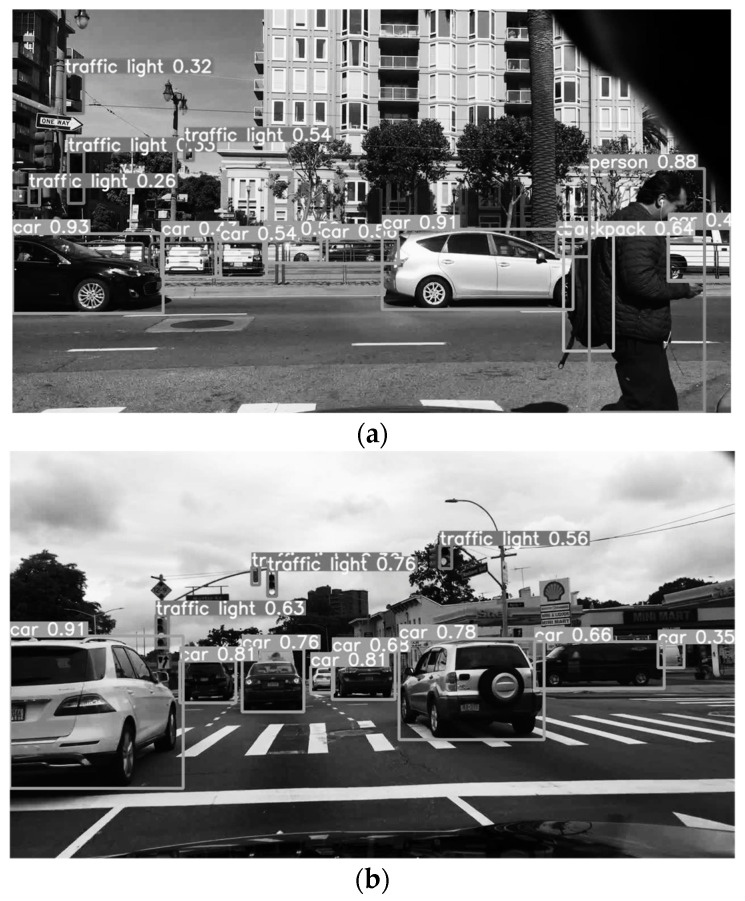

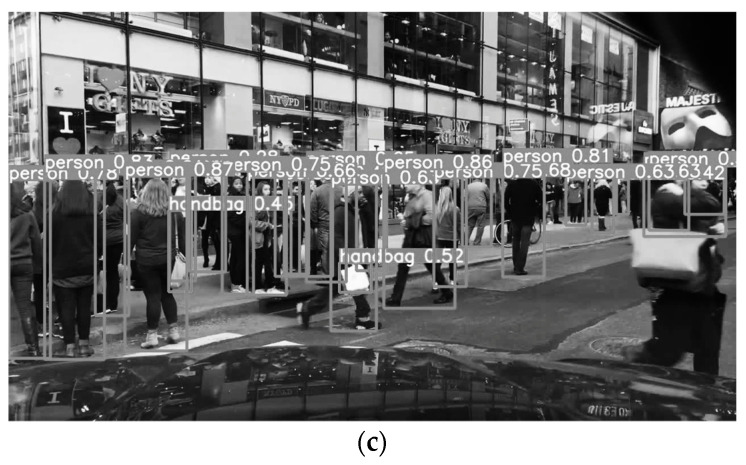
Improved algorithm to recognize the result graph: (**a**) scenario one; (**b**) scenario two; (**c**) scenario three.

The improved model and the traditional YOLOv5 algorithm model were trained and tested using the data set. Figure 11 shows the recognition results of the modified algorithm, which can effectively identify pedestrians and vehicles in complex road conditions with a low missed detection rate, and provide correct traffic warning for commercial vehicles.

Table 2 shows the detection results under different algorithms. The accuracy rate is the ratio between the number of targets correctly predicted by the model and the total number of predicted targets during object detection. It is used to measure the detection accuracy of the model, that is, how many objects the model can correctly detect. The FPS is used to measure the real-time performance of the model, that is, how many frames of video the model can process per unit time, and the mAP is often used to evaluate the detection performance of a model over the entire data set. Compared with traditional algorithms, the accuracy rate of the improved algorithm is increased by 2.59%, 2.05% and 3.93%, the mAP is increased by 2.37%, 6.97% and 1.56%, and the frame rate is increased by 22.5%, 16.5% and 3.68%. The experimental results show that the improved algorithm has an improved accuracy and precision compared with the traditional algorithm.

In order to further verify the performance of the algorithm and quantitatively analyze each module, four groups of ablation experiments are designed in this paper. The experimental data sets, the training data set and the val data set of the Berkeley data set are selected, and the experimental results are shown in Table 3.

The FEM module in Figure 7 consists of an MF module and CPM module. The MF module represents the introduction of edge features and infrared features, and the CPM module represents the introduction of the composite prediction mechanism. mAP_0.5 indicates that the IoU threshold of the map is 0.5, and mAP_0.5:0.95 indicates that the IoU value of the map is increased from 0.5 to 0.95 in increments of 0.05. It can be seen from the table data that after adding all the modules to the Yolov5 algorithm, the accuracy increased by 3.78%, the mAP_0.5 increased by1.54% and the mAP_0.5:0.95 is increased by 10.44%.

### 5.3. Real Car Experiment

**Figure 12:**
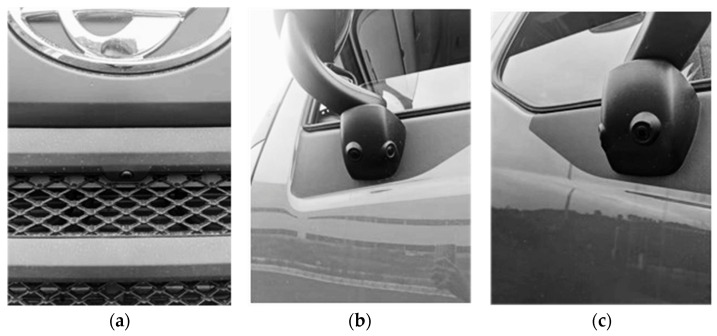
Related equipment installation diagram: (**a**) front-view camera installation position; (**b**) left camera installation position; (**c**) right camera installation position.

**Figure 13:**
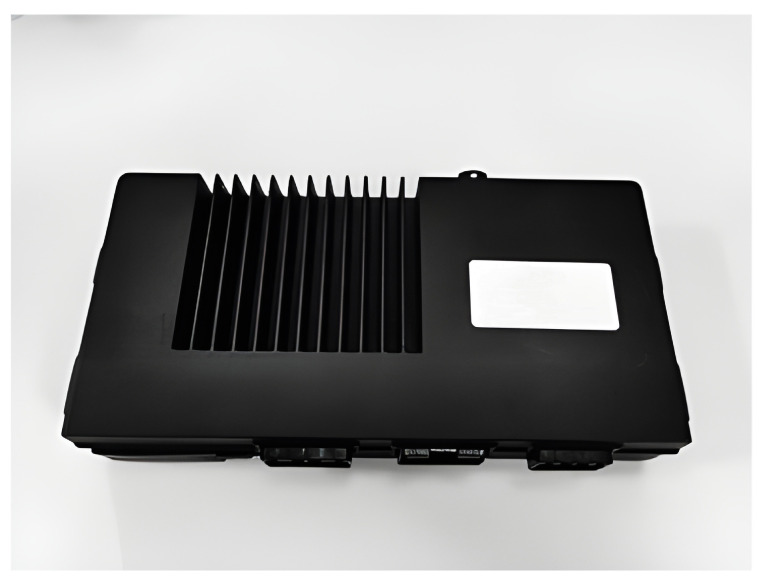
Sensing system host diagram.

In order to verify whether the improved algorithm can fulfill the requirements of surround perception and target recognition and early warning, some real vehicle experiments are needed. The commercial vehicle of Dongfeng Liuzhou Automobile Co., Ltd, produced in Liuzhou, China is used to collect relevant real vehicle data according to the experimental regulations. Figure 12 shows the installation of the relevant equipment. The circumnavigation cameras are installed in front of the vehicle and under the rearview mirrors on both sides of the vehicle. Figure 13 shows the host diagram of the sensing system. The data collected by the body camera are collected and processed by the host of the system.

**Figure 14:**
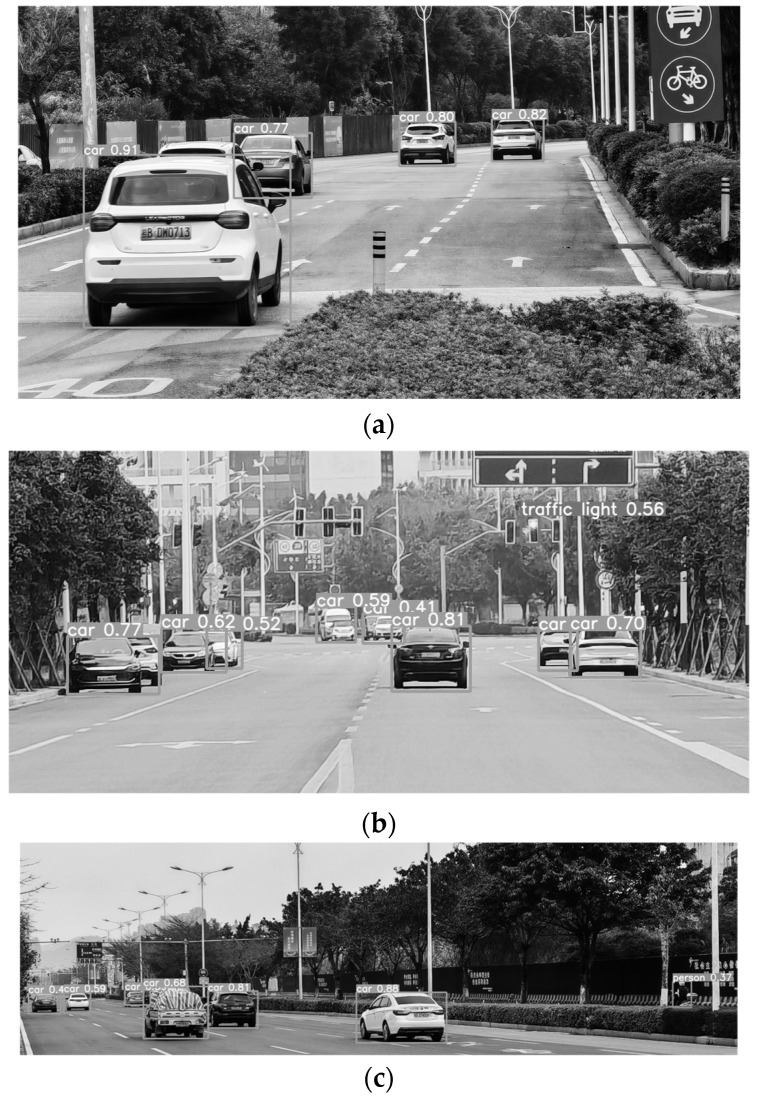
Real car identification renderings: (**a**) road condition one; (**b**) road condition two; (**c**) road condition three.

Figure 14 is the detection result of the improved algorithm. The three diagrams show the recognition effect of the algorithm under three road conditions. It can be seen that when external vehicles are in a dynamic state, they can be recognized effectively when the distance is relatively close and when the distance is relatively far, meeting the requirements of an early traffic safety warning. Table 4 shows the identification conditions of the experimental vehicle under various road conditions. Under the same road scene and mileage, the number of missed identifications in urban road conditions is reduced by 33%, and the accuracy rate is increased by 1.81%; under national road conditions, the number of missed identifications is reduced by 44%, and the accuracy rate is increased by 2.69%. Compared with the traditional algorithms, the proposed algorithm has certain advantages in terms of the accuracy and recognition accuracy.

## 6. Conclusions

This paper proposes a commercial vehicle surround sensing method based on multi-features and an improved YOLOv5 algorithm. In this method, the edge features and infrared features of the target region are extracted from the images collected by the body camera, and the low-dimensional SIFT feature point set is generated for image registration. Then, the images that can be stitched are fused. The improved YOLOv5 algorithm is adopted to recognize the fused images, and the target of the surround perception is completed after the preset targets are identified.

The experimental results show that compared with the traditional image registration algorithm, the proposed method has improved the registration time and accuracy. The image stitching accuracy is increased by 17% on average, the time consumption is reduced by 89% on average, and the target recognition process is improved by 2.86% on average compared with the improved method. The real car test also shows that the method can effectively perceive the surroundings of trucks, accurately identify the targets, and the missed warning rate and false warning rate are reduced to some extent.

In the future, there will be further research on the construction and recognition of on-board panoramas.

## Figures and Tables

**Table 1:** Experimental data of three sets of images.

Title 1	NMP (Logarithm of Matching)	MA (Correct Matching Rate)	T (Time of Image Matching)	Image Set
SIFT	845	72.3%	12.46	First set of images
SIFT	1713	68.4%	23.44	Second set of images
SIFT	1083	69.5%	14.76	Third set of images
Improved SIFT	714	80.6%	10.39	First set of im-ages
Improved SIFT	1420	79.1%	16.26	Second set of images
Improved SIFT	896	84.6%	11.15	Third set of images
SIFT–Harris	352	87.1%	4.89	First set of images
SIFT–Harris	909	89.2%	13.55	Second set of images
SIFT–Harris	395	82.7%	5.02	Third set of images
Textual algorithm	164	96.8%	0.90	First set of images
Textual algorithm	243	95.1%	1.55	Second set of images
Textual algorithm	179	97.6%	1.30	Third set of images

**Table 2:** Comparison of algorithm detection results.

Algorithm	Accuracy Rate	mAP	FPS
Yolov4	0.852	0.764	45
Yolov5m	0.928	0.885	52.9
Yolov5l	0.933	0.847	55.6
Yolov5s	0.916	0.892	62.5
Textual algorithm	0.952	0.906	64.8

**Table 3:** Ablation experiment results.

Algorithm	Accuracy Rate	mAP_0.5	mAP_0.5:0.95
Yolov5s	0.916	0.892	0.583
Yolov5s + MF	0.923	0.897	0.598
Yolov5s + CPM	0.937	0.902	0.624
Yolov5s + MF + CPM	0.952	0.906	0.651

**Table 4:** Road test results.

Road	Algorithm	Mileage	Total Identification Number	Missing Identification Number	Accuracy Rate
Town	Traditional algorithm	67	93	3	93.61%
	Textual algorithm	67	85	2	95.42%
National road	Traditional algorithm	302	134	9	92.83%
	Textual algorithm	302	157	5	95.52%

## Data Availability

Due to the nature of this research, participants of this study did not agree for their data to be shared publicly, so supporting data are not available.
